# Artificial intelligence in the diagnosis of multiple sclerosis using brain imaging modalities: A systematic review and meta-analysis of algorithms

**DOI:** 10.1097/MD.0000000000044493

**Published:** 2025-09-19

**Authors:** Reza Darrudi, Azamossadat Hosseini, Hassan Emami, Arash Roshanpoor, Mohammad Ali Nahayati

**Affiliations:** a Faculty of Paramedical Sciences, Shahid Beheshti University of Medical Sciences, Tehran, Iran; b Department of Health Information Technology and Management, School of Allied Medical Sciences, Shahid Beheshti University of Medical Sciences, Tehran, Iran; c Department of Computer, Yadegar-e-Imam Khomeini (RAH), Shahre Rey Branch, Islamic Azad University, Tehran, Iran; d Department of Neurology, Ghaem Hospital, Mashhad University of Medical Sciences, Mashhad, Iran.

**Keywords:** artificial intelligence, diagnosis, medical imaging, meta-analysis, multiple sclerosis, systematic review

## Abstract

**Background:**

Multiple sclerosis (MS) diagnosis remains challenging due to its heterogeneous clinical manifestations and the absence of a definitive diagnostic test. Conventional magnetic resonance imaging, while central to diagnosis, faces limitations in specificity and inter-rater variability. Artificial intelligence offers promising solutions for enhancing medical imaging analysis in MS, yet its efficacy requires systematic validation.

**Methods:**

This systematic review and meta-analysis followed Preferred Reporting Items for Systematic Review and Meta-Analysis guidelines. We searched Embase, PubMed, Web of Science, Scopus, Google Scholar, and gray literature (inception to January 5, 2025) for case–control studies applying AI to magnetic resonance imaging-based MS diagnosis. A random-effects model pooled sensitivity, specificity, and accuracy. Heterogeneity was assessed via the *Q*-statistic and *I*². Meta-regression evaluated pixel count impact.

**Results:**

Meta-analysis revealed pooled sensitivity, specificity, and accuracy of 93%, 95%, and 94%, respectively, showcasing the efficacy of AI models in MS diagnosis. Additionally, meta-regression analysis showed no significant correlation between the number of pixels and diagnostic performance parameters. Sensitivity analysis confirmed the robustness of results, while publication bias assessment indicated no evidence of bias.

**Conclusion:**

AI-based algorithms show promise in augmenting traditional diagnostic approaches for MS, offering accurate and timely diagnosis. Further research is warranted to standardize AI methodologies and optimize their integration into clinical practice. This study contributes to the growing evidence supporting AI’s role in enhancing diagnostics and patient care in MS.

## 1. Introduction

Multiple sclerosis (MS) is a chronic autoimmune disease of the central nervous system, specifically the brain and spinal cord.^[1]^ The exact cause of MS is not fully understood, but it is believed to involve a combination of genetic and environmental factors.^[2,3]^

Based on the most recent estimates, the global population living with MS stands at 2.8 million, equating to a prevalence rate of 36 per 100,000 individuals. Notably, the prevalence varies significantly across different geographical regions, with Europe and the Americas reporting the highest rates at 133 and 112 per 100,000 people, respectively. In contrast, Africa exhibits a notably lower prevalence at 5 per 100,000, while Southeast Asia registers a slightly higher rate of 9 per 100,000 individuals.^[4]^ MS is more common in women than men, with a female-to-male ratio of approximately 3:1. The disease typically affects young adults, with the onset of symptoms occurring between the ages of 20 and 40.^[5–7]^

The diagnosis of MS poses a complex challenge due to the absence of a single definitive test for confirmation. This complexity is particularly evident in cases featuring atypical symptoms or progressive disease, further complicating the diagnostic process.^[8]^ It is important to note that the diagnosis of MS often relies on ruling out other conditions that might produce similar symptoms, a process known as a differential diagnosis.^[9]^

Magnetic resonance imaging (MRI) plays a crucial role in the diagnosis of MS.^[10]^ MRI stands out as the preferred imaging modality for both diagnosing MS and tracking the progression of the disease. Specifically, MRI scans of the brain and spinal cord are instrumental in visualizing MS-related effects, including the detection of lesions or scarring within the central nervous system.^[11]^ The 2017 McDonald criteria state that in order to diagnose MS, there needs to be reasonable clinical suspicion, along with supportive MRI and paraclinical evidence.^[12]^ MRI can support and substitute clinical information for MS diagnosis, allowing for earlier and more accurate diagnosis and, consequently, earlier treatment.^[13]^ Despite its utility, MRI interpretation for MS faces challenges: lesions can mimic other conditions (e.g., small vessel disease [SVD], aging); variations in scanner protocols impede consistency; poor correlation between lesion burden and clinical symptoms complicates prognostication. AI addresses these gaps by automating lesion quantification and detecting subtle patterns beyond human capability.

Artificial intelligence (AI) has demonstrated remarkable promise in supporting the diagnosis, prognosis, and monitoring of MS. The integration of AI-based algorithms into healthcare presents a significant opportunity to enhance diagnostic accuracy, refine treatment approaches, and improve overall patient outcomes for MS.^[14]^ One of the most significant applications of AI in MS is the analysis of medical imaging data, such as MRI.^[15]^ AI techniques, particularly machine learning (ML) and deep learning, have been used to improve the diagnosis of MS by analyzing MRI scans.^[16]^ AI has also been used to better understand the cognitive phenotypes of MS patients, which can help in the development of more personalized treatment strategies.^[17]^

Several recent studies have highlighted the growing role of AI in the diagnosis of MS through the analysis of medical imaging data.^[18]^ Furthermore, AI has the potential to optimize treatment strategies and enhance patient outcomes for MS. The aim of the current systematic review focuses on AI based on brain imaging modalities in the diagnosis of MS.

## 2. Materials and methods

The present study adheres to the Preferred Reporting Items for Systematic Review and Meta-Analysis guidelines.^[19]^ This review was not prospectively registered in PROSPERO. We acknowledge this limitation and have provided full methodological transparency to mitigate bias.

### 2.1. Information sources, search strategy

We conducted an extensive literature search across multiple databases, including Embase, PubMed, Web of Science, and Scopus. Additionally, we expanded our search to include Google Scholar, reviewed the initial 10 pages of results, and explored gray literature sources to ensure comprehensive coverage of relevant articles. Our primary objective was to identify research studies employing AI models for the diagnosis of MS. The exclusion criteria were defined to delete studies that failed to report essential outcomes such as sensitivity and specificity. Studies were discounted if they lacked clear reporting of training and test datasets. Eligible studies were case-control designs published in English. Review articles, non-imaging studies, and nondiagnostic AI applications (e.g., prognosis) were excluded. Last search was performed to investigate articles from inception to January 5, 2025. We employed a detailed search strategy tailored to each database, using a combination of Medical Subject Headings terms and free-text terms such as “artificial intelligence,” “neural network,” “machine learning,” “deep learning,” and “Multiple sclerosis”. Manual searches of references and gray literature supplemented database queries. The strategy was adjusted to accommodate each database’s specific indexing systems and syntaxes. The bibliometrics network map of AI and MS is illustrated in Figure S1, Supplemental Digital Content, https://links.lww.com/MD/P938.

### 2.2. Data extraction and quality assessment

Two authors (RD and HE) conducted an independent review of abstracts and titles to identify studies for thorough full-text examination. Subsequently, data extraction and quality assessment were performed independently following the initial screening phase. Any discrepancies that arose during this process were resolved through consultation between the 2 authors. The quality of the studies was evaluated using the Quality Assessment of Diagnostic Accuracy Studies-2 (QUADAS-2) tool, comprising 12 questions designed to assess the risk of bias and applicability across 4 domains: patient selection, appropriateness of the index test, reference standard, and flow and timing. Each question necessitated a response indicating high, low, or unclear risk. Data extracted from each study encompassed various parameters, including the first author’s name, year of publication, country of research, study design, the total number of participants, types of AI classifiers employed (e.g., convolutional neural network [CNN], multilayer perceptron, artificial neural network), modality type, and statistics related to sensitivity, specificity, and accuracy. A meticulous quality assessment was conducted utilizing risk-of-bias visualization to ascertain adherence to the QUADAS-2 criteria.^[20]^

### 2.3. Statistical analysis

In this study, we pooled the outcome data utilizing a random-effects model. Heterogeneity among the included studies was assessed using the *Q* statistic and *I*-squared test (*I*^2^), with *I*^2^ categorized as low (0–50%), moderate (51–75%), or high (>75%). A meta-regression analysis was carried out to explore the correlation between the number of pixels and diagnostic performance parameters. Additionally, sensitivity analyses were conducted using a one-by-one study removal method to evaluate the robustness of the results. To assess potential publication bias, Egger test was employed in conjunction with funnel plot inspection. Statistical significance was determined at *P* < .05, with a 95% confidence interval (95% CI) serving as the effect size in the analysis. The data analysis was conducted using Comprehensive Meta-analysis software version 3.0 (Biostat, Inc., Englewood).

## 3. Results

### 3.1. Study selection

**Figure 1.**
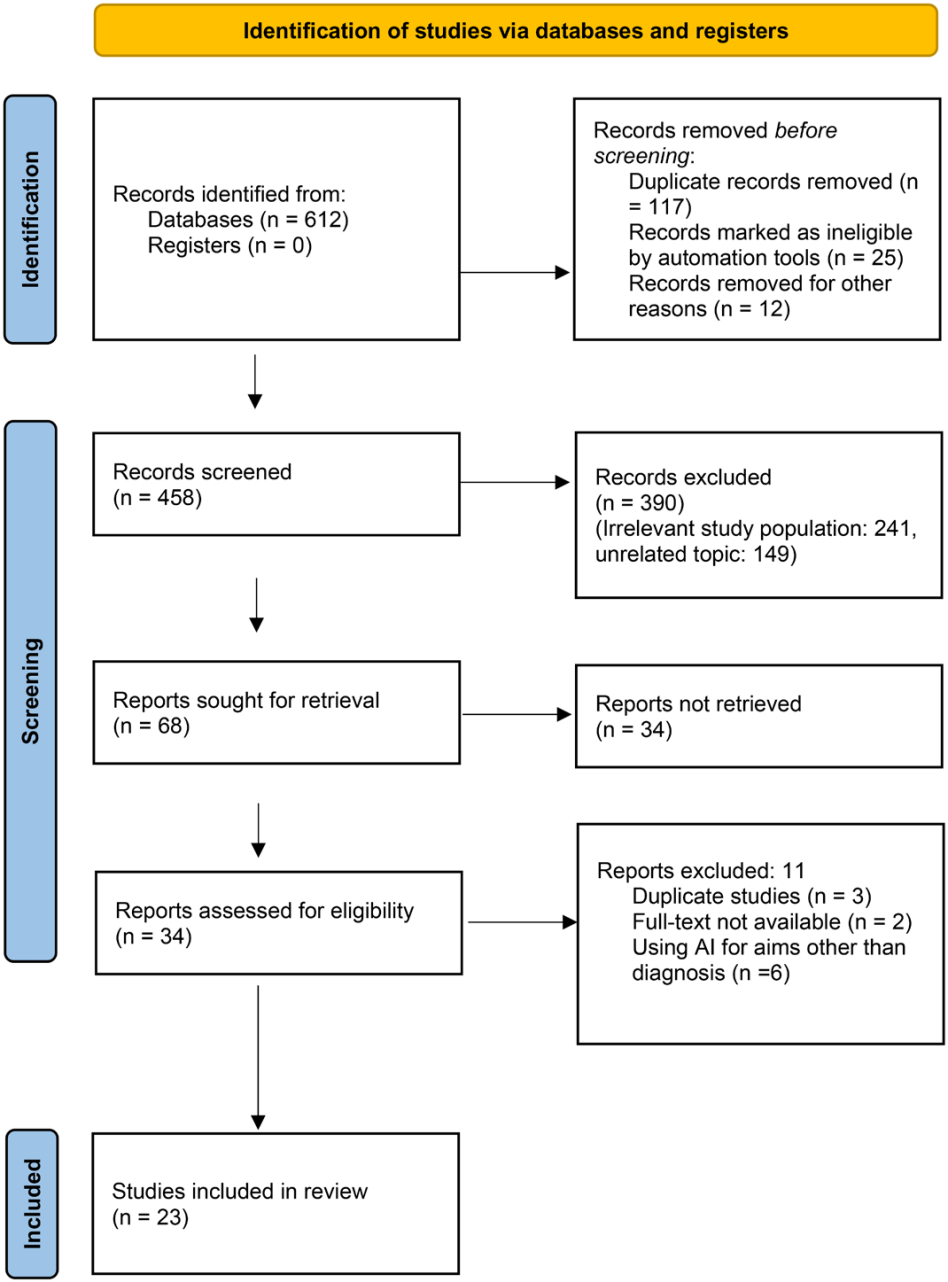
Flow diagram of search methodology and literature selection process.

Following the Preferred Reporting Items for Systematic Review and Meta-Analysis 2020 guidelines, the systematic review and meta-analysis commenced with the initial identification of 612 articles. After eliminating duplicates, 458 articles underwent the screening stage. The reasons for excluding 390 records were: irrelevant study population (241) and unrelated topic (149). Subsequently, the full texts of 68 articles underwent a comprehensive examination for inclusion. Among these, 3 articles were removed due to duplicate studies, 6 due to the use of AI for purposes other than diagnosis, and 2 articles due to full-text availability. As a result, 23 articles were included for qualitative synthesis. The entire selection process is visually presented in Figure 1.

### 3.2. Study characteristics

The characteristics of the included studies^[21–42]^ are outlined in Table 1. The selected studies span from 2016 to 2023. Geographically, eleven studies were from China, 4 were from Iran, 3 from Germany, and one each was from South Korea, Italy, France, Russia, and Türkiye. The total sample consisted of 2904 participants (MS: 1252; controls: 1652). Variety used AI methods in the included studies presented in Figure S2, Supplemental Digital Content, https://links.lww.com/MD/P938.

**Table 1:** Basic information of included studies.

Author, year	Country	Design	Sample size	MS group	Control group	Algorithm	Modality	SEN	SPE	ACC
Seok et al^[41]^	South Korea	Case–control	156	86	70	CNN	MRI	0.77	0.75	0.76
Rocca et al^[29]^	Italy	Case–control	268	70	198	CNN	MRI	NR	0.98	0.99
Iswisi et al^[25]^	Türkiye	Case–control	8887 pixels	NR	NR	FCM	MRI	0.90	0.93	0.94
Zhang et al^[38]^	China	Case–control	72	34	38	SWE, ML	MRI	0.96	0.99	0.98
Wang et al^[34]^	China	Case–control	72	34	38	BWT, PCA	MRI	0.97	0.98	0.98
Neeb^[28]^	Germany	Case–control	97	36	61	NN	MRI	0.92	0.86	0.89
Marzullo et al^[27]^	France	Case–control	114	66	48	CNN	MRI	0.93	NR	NR
Han and Hou^[24]^	China	Case–control	64	38	26	ANN, NN	MRI	0.92	0.92	0.92
Siar and Teshnehlab^[30]^	Iran	Case–control	127	47	80	CNN	MRI	0.95	0.95	0.97
Zhang et al^[15]^	China	Case–control	38	19	19	CNN, GAP	MRI	NR	NR	0.98
Zhang et al^[39]^	China	Case–control	64	38	26	CNN	MRI	0.98	0.98	0.98
Lopatina et al^[26]^	Germany	Case–control	132	66	66	ML, NN, CNN	MRI	NR	NR	0.95
Zhou and Shen^[40]^	China	Case–control	64	38	26	GLCM, BBO, NN	MRI	0.93	0.93	0.93
Zhang^[38]^	China	Case–control	72	34	38	CED, MBD, NN	MRI	NR	0.98	0.98
Wu and Lopez^[36]^	China	Case–control	67	33	34	PC, LR	MRI	NR	NR	0.90
Vatian et al^[42]^	Russia	Case–control	19	9	10	CNN	MRI	NR	NR	0.88
Soltani and Nasri^[31]^	Iran	Case–control	72	34	38	CNN	MRI	0.99	0.99	0.99
Fooladi et al^[23]^	Iran	Case–control	60	30	30	ANN, MLP	MRI	0.93	0.88	0.90
Eitel et al^[22]^	Germany	Case–control	147	76	71	CNN, ML	MRI	0.77	NR	0.88
Azarmi et al^[21]^	Iran	Case–control	20	8	12	ML	MRI	0.88	NR	0.95
Wang et al^[33]^	China	Case–control	64	38	26	CNN	MRI	0.98	0.99	0.99
Wang and Zhang^[20]^	China	Case–control	72	38	34	TLBA	MRI	0.98	0.98	0.98
Wang et al^[32,33]^	China	Case–control	64	38	26	FRFE, MLP	MRI	0.97	0.97	0.97

ACC = accuracy; ANN = artificial neural network, BBO = biogeography-based optimization; BWT = biorthogonal wavelet transform; CED = Canny Edge Detector; CNN = convolutional neural network; FCM = Fuzzy C-means; FREF = fractional Fourier entropy; GAP = global average pooling; GLCM = grey-level cooccurrence matrix; LR = logistic regression; MBD = Minkowski Bouligand Dimension; ML = machine learning; MLP = multilayer perceptron; MRI = magnetic resonance imaging; NN = neural network; PC = principal component; SEN = sensitivity; SPE = specificity; SWE = stationary wavelet entropy; TLBA = transfer learning-based approach.

### 3.3. Quality assessment

**Figure 2.**
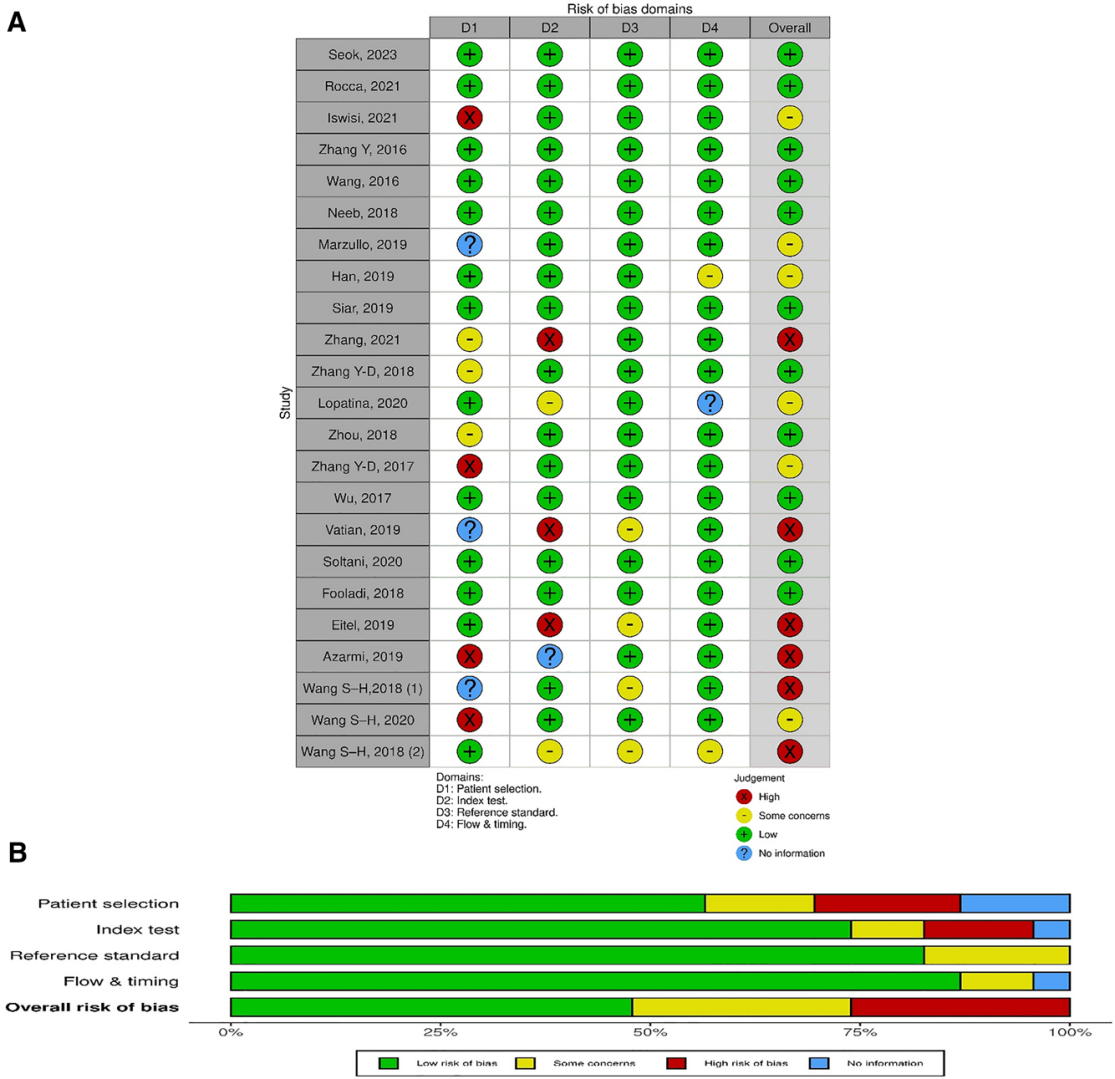
Visualization of risk of bias based on the QUADAS scores. (A) Displays the quality assessment, depicting the individual risk-of-bias domains for each study included in the meta-analysis. (B) Presents the overall scores of the studies included in the meta-analysis. QUADAS = Quality Assessment of Diagnostic Accuracy Studies.

The quality assessment conducted according to QUADAS-2 revealed that the majority of studies exhibited a low risk of bias and had no apparent applicability concerns. Importantly, in terms of the diagnostic process, 11 studies presented a low risk, 6 studies presented some concerns, and 6 studies presented a high risk of bias. The quality assessment of the included studies is illustrated in Figure 2.

### 3.4. Main results

#### 3.4.1. Sensitivity

**Figure 3.**
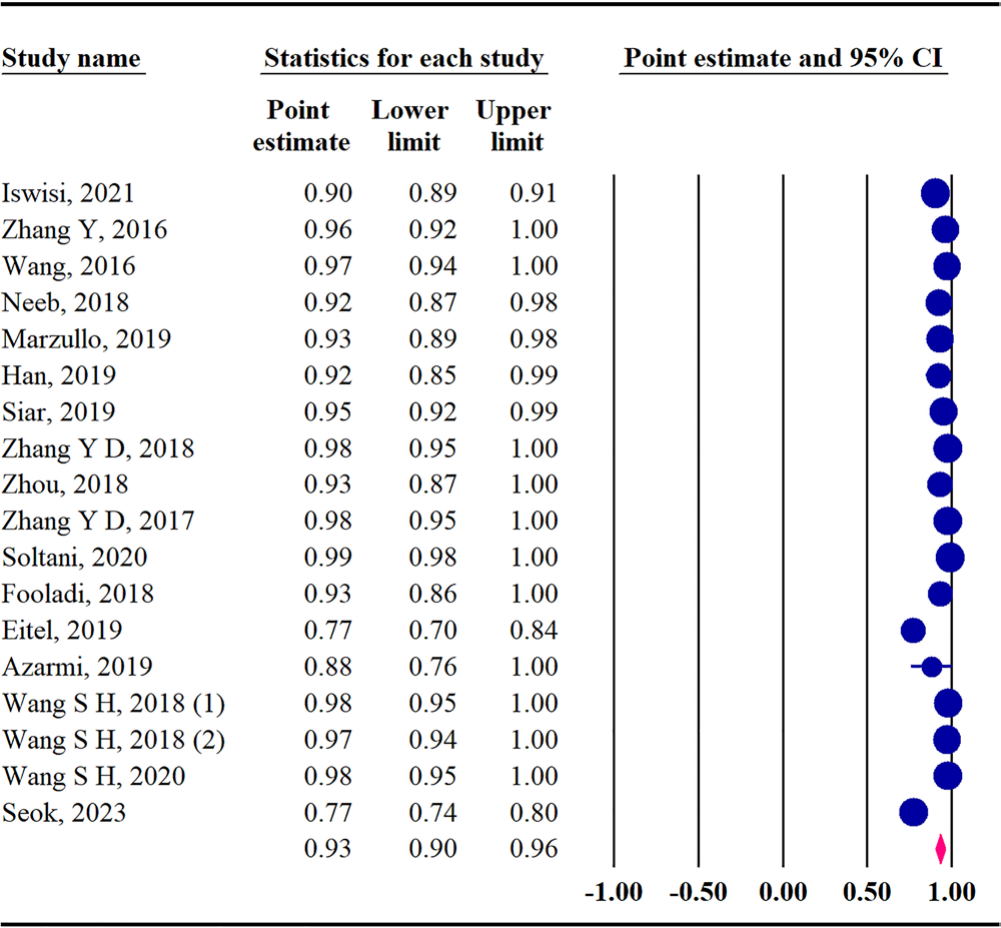
The forest plot of pooled sensitivity.

The pooled sensitivity among all studies was 93% (95% CI: 90–96; *Q* = 310.02, *P* < .001, *I*^2^ = 94.51%) (Fig. 3).

#### 3.4.2. Specificity

**Figure 4.**
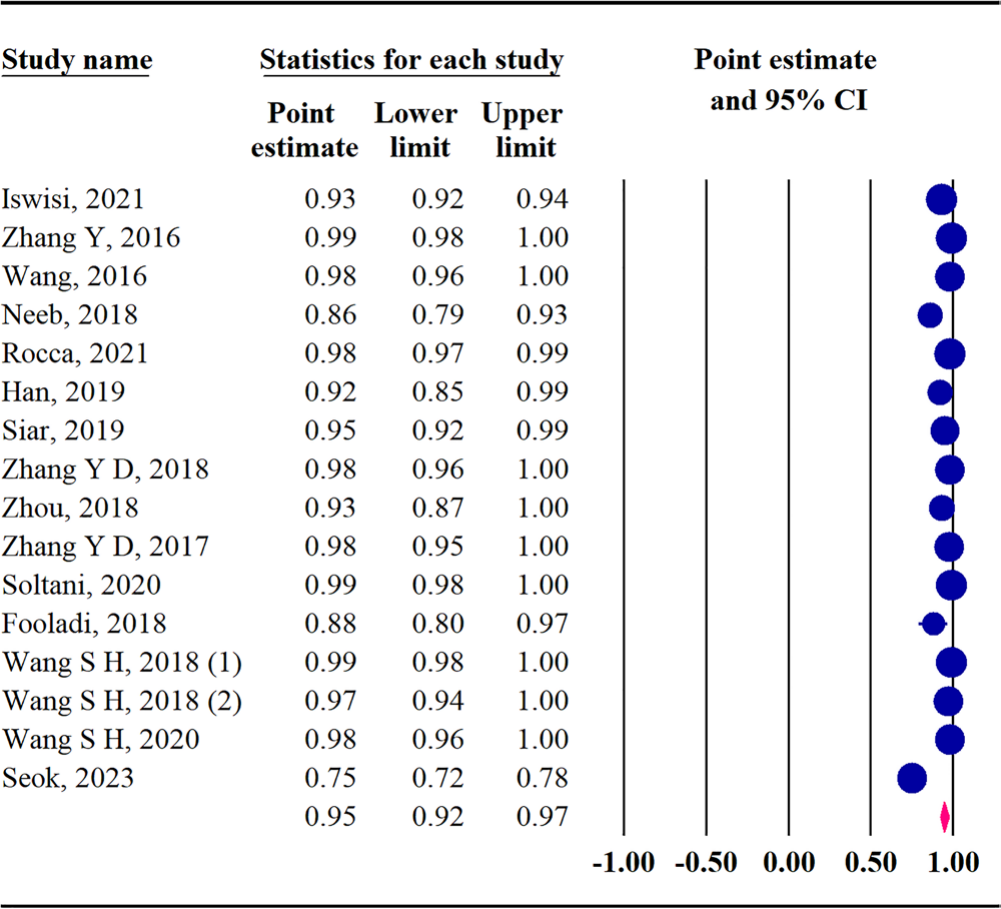
The forest plot of pooled specificity.

Based on the included studies, the pooled specificity was 95% (95% CI: 92–97; *Q* = 388.78, *P* < .001, *I*^2^ = 96.14%) (Fig. 4).

#### 3.4.3. Accuracy

**Figure 5.**
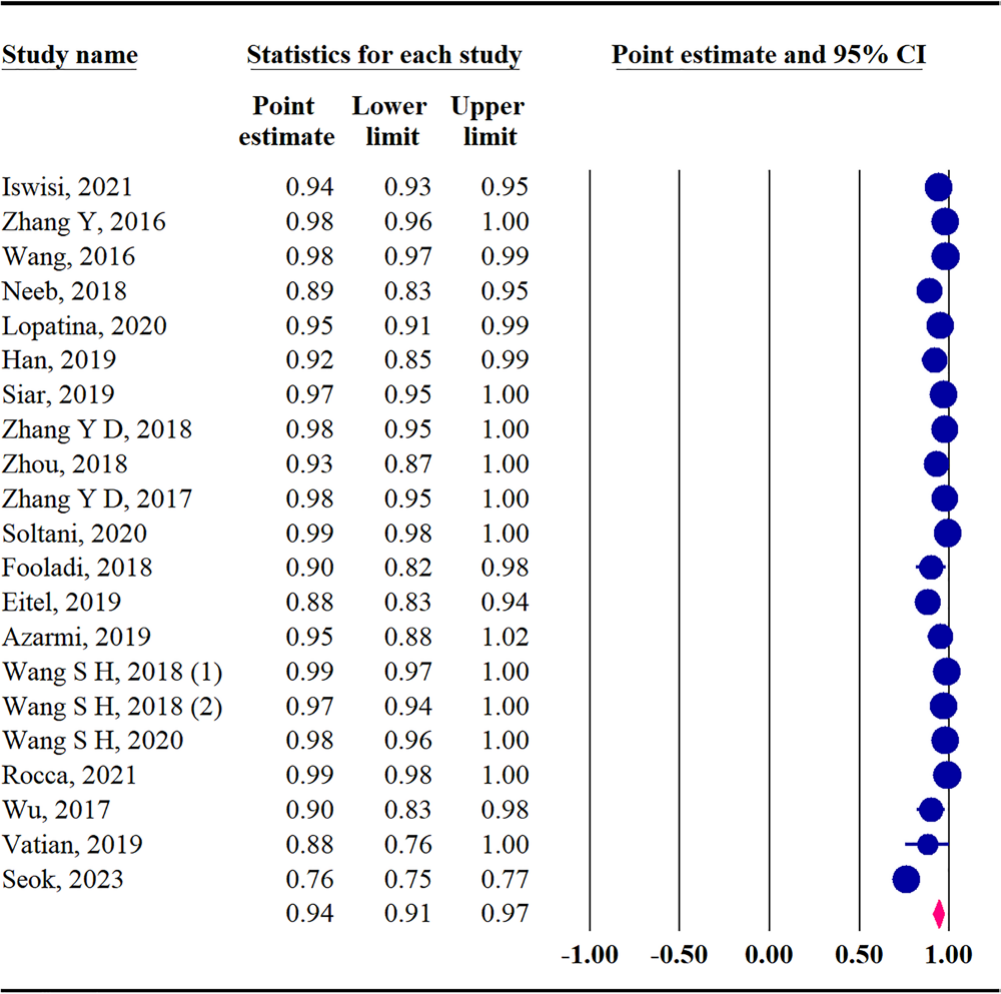
The forest plot of pooled accuracy.

Our analysis showed 94% accuracy among studies that used AI in MS diagnosis (95% CI: 91–97 *Q* = 1048.64, *P* < .001, *I*^2^ = 98.09%) (Fig. 5).

#### 3.4.4. Meta-regression

**Figure 6.**
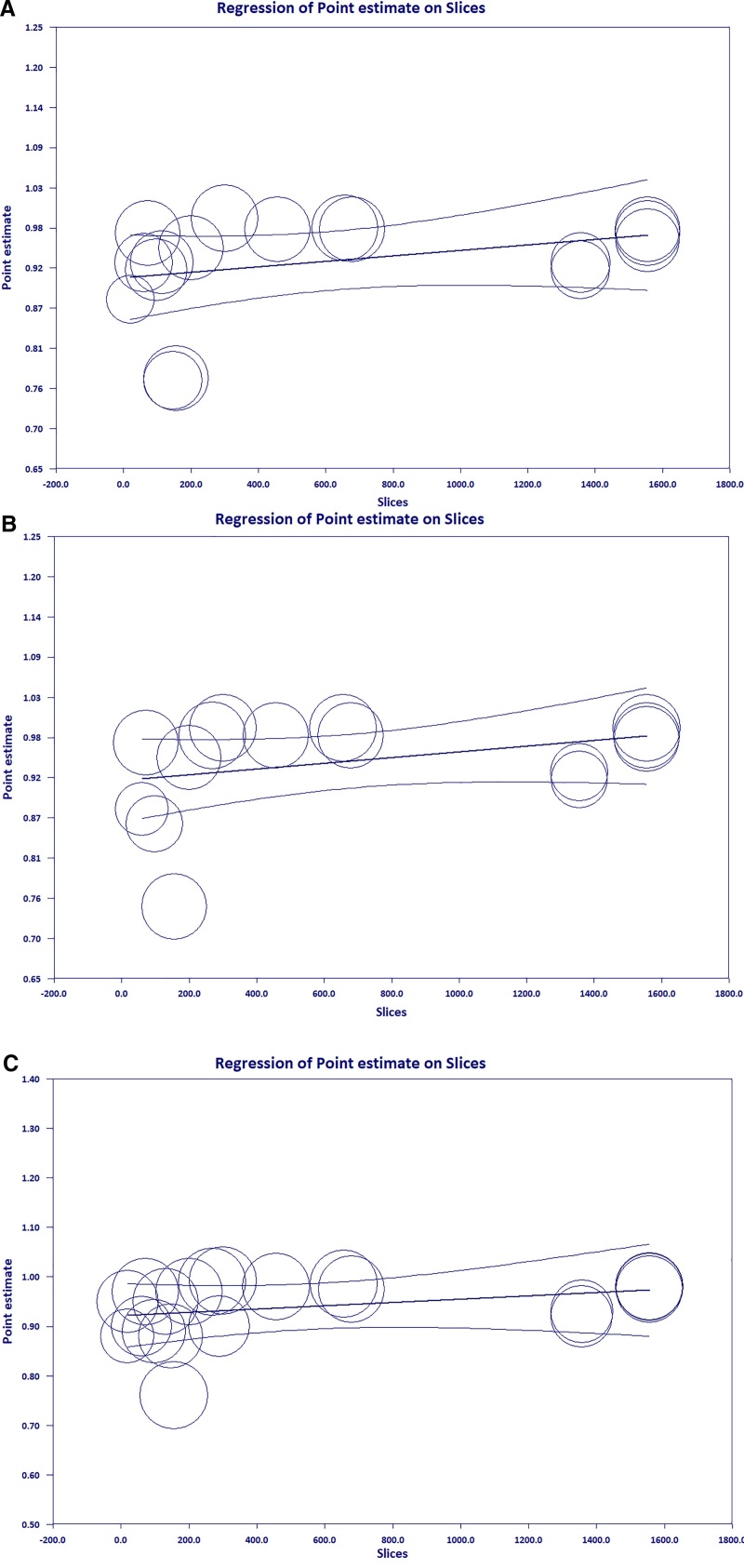
The results of meta-regression analysis based on the number of pixels and sensitivity (A), specificity (B), and accuracy (C).

The meta-regression analysis aimed to investigate the correlation between the number of pixels and diagnostic performance parameters. Results indicated no statistically significant difference between the outcome of the meta-analysis and an increased number of pixels for sensitivity (coefficient = 0.0001, *P* = .178), specificity (coefficient = 0.0001, *P* = .131), and accuracy (coefficient = 0.0001, *P* = .319) (see Fig. 6).

#### 3.4.5. Sensitivity analysis

Sensitivity analysis was performed by systematically removing each study to evaluate its impact on the overall outcome. The results of the sensitivity analysis demonstrated no significant alteration in the pooled result upon the removal of individual studies.

#### 3.4.6. Publication bias

**Figure 7.**
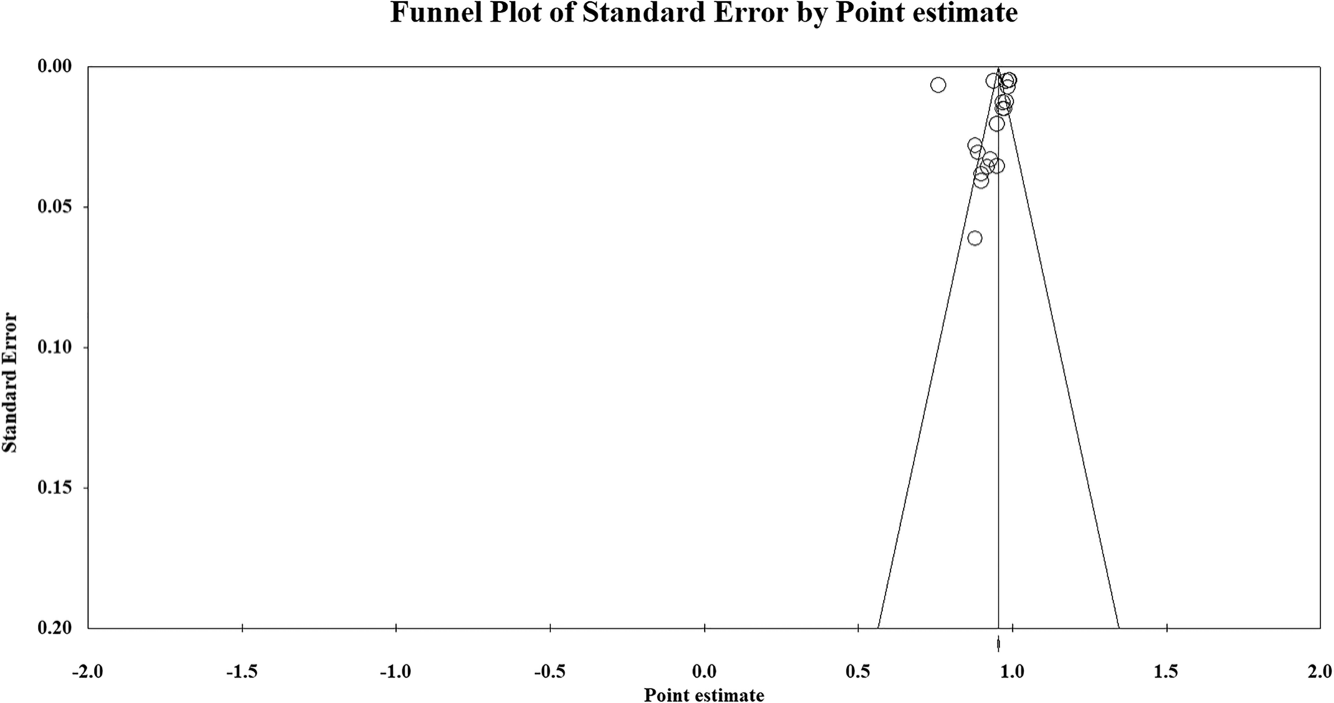
Funnel plot of the publication bias assessment.

Analysis of the dataset revealed no indications of publication bias, as evidenced by Egger test with a *P*-value of .635 (see Fig. 7).

## 4. Discussion

The current systematic review focuses on AI based on brain imaging modalities in the diagnosis of MS.

Punctate lesions observed in neuroimaging can arise from various pathologies, notably SVDs and MS. Understanding these differences is crucial for accurate diagnosis and management. SVD lesions, known as leukoaraiosis, appear as scattered hyperintensities primarily in the white matter, often linked to chronic ischemia. SVD lesions are visible on T2-weighted and fluid-attenuated inversion recovery sequences and may progress to confluent areas and are associated with microhemorrhages.^[43,44]^ SVD lesions are linked to cognitive decline and increased stroke risk, serving as markers for vascular cognitive impairment.^[44]^ MS lesions are T2 hyperintensities found in juxtacortical and periventricular areas, representing demyelination rather than ischemia. MS lesions are typically well-defined and may enhance with contrast during active phases. Cortical gray matter involvement is also common.^[45,46]^ MS lesions correlate with neurological symptoms like motor deficits and cognitive changes, crucial for diagnosis and monitoring.^[46,47]^

### 4.1. MS versus SVD differentiation

MS lesions typically appear as well-defined T2 hyperintensities in juxtacortical/periventricular regions, often enhancing with contrast during active phases. In contrast, SVD lesions manifest as scattered white matter hyperintensities linked to ischemia, rarely showing contrast enhancement. AI algorithms (e.g., CNNs) excel by quantifying spatial distribution, edge sharpness, and enhancement patterns (key differentiators overlooked in manual assessment).

### 4.2. Algorithm-specific roles

CNNs dominated included studies (15/23), leveraging hierarchical feature extraction for lesion segmentation. Algorithms like Fuzzy C-means and transfer learning showed higher specificity in smaller datasets by reducing noise sensitivity. Quantitative approaches (e.g., texture analysis via grey-level cooccurrence matrix) complemented qualitative assessment by standardizing lesion characterization across scanners.

The results of our study show that pooled sensitivity among all studies for the diagnosis of MS was 93%. AI models based on MRI have high sensitivity in the diagnosis of MS because they can accurately detect and quantify white matter lesions, which are a hallmark of the disease. AI algorithms can automate lesion segmentation and quantification, reducing the manual effort and time required by radiologists.^[14]^ Research has consistently demonstrated the capacity of AI models to assist clinicians in achieving precise diagnoses of MS, particularly exhibiting high sensitivity, particularly when leveraging MRI data.^[48]^ Furthermore, AI models possess the capability to forecast clinical disability and assess the long-term benefits and safety of treatments, rendering them indispensable tools in the management of MS. The findings highlight that AI models are not only capable of predicting disability related to active disease but also play a crucial role in forecasting long-term disability progression in MS. This dual capability enhances the overall management of MS, making AI an indispensable tool in clinical practice.

The pooled specificity for the diagnosis of MS, as indicated by our study, stands at 95%. AI algorithms exhibit remarkable accuracy in identifying and delineating new active lesions, facilitating swift and consistent lesion identification. This efficiency significantly reduces the manual workload and time burden on radiologists, ultimately contributing to enhanced specificity in MS diagnosis.^[49]^ MRI imaging provides comprehensive anatomical and structural details of the brain and spinal cord, enabling the detection of lesions, atrophy, and other abnormalities characteristic of MS. ML models can undergo training to identify intricate patterns within these images, often imperceptible to the human eye, enhancing diagnostic accuracy, and precision.^[50]^ ML models can perform quantitative analysis on MRI data, allowing for the measurement of subtle changes in tissue characteristics. This quantitative approach can enhance the accuracy of distinguishing normal from abnormal tissue, improving specificity.^[51]^ AI models excel at extracting relevant features from complex datasets. In the case of MRI, these features may include the size, shape, and location of lesions, as well as other subtle patterns that are indicative of MS.^[52]^

The result of the meta-analysis showed 94% accuracy among studies that used AI in MS diagnosis. MRI provides detailed and high-resolution images of the brain and spinal cord.^[53]^ It can detect and visualize the characteristic lesions associated with MS.^[54]^ These lesions, also known as plaques or scars, appear as areas of abnormal signal intensity and can be seen in specific regions of the central nervous system.^[55]^ MRI is sensitive to changes in water content and tissue composition.^[56]^ In MS, there is inflammation, demyelination (loss of the protective covering of nerve fibers), and sometimes axonal damage.^[57]^ These changes affect the water content and composition of tissues, making them detectable by MRI. Different MRI sequences, such as T1-weighted, T2-weighted, and fluid-attenuated inversion recovery, provide complementary information about the tissues.^[58]^ This multi-sequence approach allows for a more comprehensive assessment of MS lesions, helping to distinguish between different stages of the disease.^[59]^ Overall, the combination of high resolution, sensitivity to tissue changes, and the ability to use contrast agents makes MRI a valuable tool for the accurate diagnosis and monitoring of MS.^[60]^

Despite all of the revolutions of the MRI, several challenges persist in accurately interpreting MRI findings to ascertain MS pathology. Despite its high sensitivity for detecting lesions, conventional MRI lacks specificity for MS pathology. Lesions observed on MRI can arise from various conditions, including other demyelinating diseases, vascular issues, and even normal aging processes.^[11,54]^ Differences in scanner types, field strengths, and imaging techniques across institutions can result in inconsistent lesion detection and characterization, affecting clinical decision-making.^[11,61]^ The expertise required for accurate MRI interpretation is often lacking among radiologists. Misinterpretation can occur without a comprehensive understanding of the patient’s clinical history.^[61]^ There is often a poor correlation between the number of lesions detected on MRI and clinical symptoms, complicating prognostication and treatment planning.^[11,62]^ While advanced modalities like diffusion tensor imaging and magnetization transfer imaging show promise in enhancing diagnostic precision, they are not yet routinely used in clinical practice due to variability in availability and expertise.^[62,63]^

To enhance the accuracy of MRI in diagnosing MS, several strategies can be implemented:

1. Adoption of advanced imaging techniques.

- *Magnetization transfer imaging*: Improves detection of myelin content, aiding in differentiating demyelination from remyelination.^[62]^
- *Diffusion tensor imaging*: Reveals microstructural changes in white matter, providing insights into neurodegeneration.^[62,64]^
- *Double inversion recovery*: Effectively detects cortical lesions often missed by standard methods.^[65]^

1. Standardization of imaging protocols.

- *Uniform protocols*: Establish standardized MRI protocols to ensure consistency in image acquisition and interpretation across institutions.
- *Field strength optimization*: Promote the use of higher field strength MRI machines (3T or greater) for better lesion visibility.^[11,66]^

1. Integration of AI.

- *Deep learning algorithms*: Utilize AI tools for automated lesion detection, reducing variability and improving diagnostic accuracy.^[64]^
- *Computer-aided diagnosis systems*: Implement CADS to assist radiologists in identifying lesions based on extensive datasets.^[64]^

1. Enhanced training for clinicians.

- *Specialized training programs*: Develop targeted training for radiologists and neurologists focused on MS imaging.
- *Interdisciplinary collaboration*: Foster collaboration between neurologists, radiologists, and researchers to improve understanding and interpretation of MRI findings.

As AI continues to transform the landscape of medical diagnostics, clinicians can effectively integrate AI tools into their practice without needing to be experts in the underlying technology.

1. Understanding AI’s role in MS diagnosis.
2. Utilizing user-friendly AI tools.
3. Training and support by participating in workshops and training sessions, and collaboration with IT specialists.
4. Interpreting AI outputs by applying sensitivity and specificity metrics and correlating clinical assessments.
5. Continuous learning and adaptation by staying updated and using feedback mechanisms.

Choosing selected AI methods over other methods, there are several advantages:

1. *High diagnostic accuracy*: The AI algorithms used in this study demonstrated a pooled sensitivity of 93% and specificity of 95% in diagnosing MS. These metrics indicate that the AI methods not only accurately identify MS but also minimize false positives, thereby enhancing diagnostic reliability compared to traditional methods that may have lower sensitivity and specificity rates.
2. *Advanced imaging analysis*: AI techniques, particularly deep learning models, excel in analyzing complex MRI data. They can automatically segment lesions and assess their characteristics more efficiently than human radiologists. This capability is crucial for:

- *Automated detection*: AI can quickly identify MS-related lesions, which reduces the time clinicians spend on image analysis.
- *Quantitative metrics*: These algorithms provide quantitative assessments of lesion burden, aiding in treatment planning and monitoring disease progression.

1. *Reduction of inter-rater variability*: Traditional MRI interpretation can vary significantly among radiologists due to differences in expertise and experience. By employing standardized AI algorithms, the variability in diagnostic outcomes can be minimized, leading to more consistent and reliable results across different clinical settings.
2. *Robustness across diverse populations*: The present study included studies from various regions and healthcare settings, demonstrating that the selected AI methods maintain reliability across diverse populations. This broad applicability is essential for clinical integration, as it ensures that these tools can be effectively utilized in different healthcare environments.
3. *Enhanced predictive capabilities*: Beyond diagnosis, AI models can predict long-term outcomes and treatment responses in MS patients. This predictive ability allows clinicians to tailor treatment strategies more effectively based on individual patient profiles, thereby improving overall patient management.
4. *Integration with clinical workflow*: The AI methods discussed are designed to complement existing diagnostic processes rather than replace them. This integration facilitates smoother adoption into clinical practice, allowing healthcare providers to leverage advanced technology while maintaining established diagnostic protocols.
5. *Evidence-based support*: The present review highlighted numerous studies demonstrating the efficacy of AI in diagnosing MS through MRI analysis. The high pooled accuracy rates (94%) reinforce the argument for using these methods over traditional approaches, which may not provide such reliable outcomes.

One of the primary limitations of this study was the insufficient information available to estimate the area under the curve accurately. Additionally, the varied methodologies employed in using AI to predict MS disease pose a challenge, making it challenging to select a single standardized method for implementation.

## 5. Conclusion

AI-based algorithms exhibit high sensitivity, specificity, and accuracy in detecting MS-related lesions on MRI, facilitating early and accurate diagnosis while streamlining the differential diagnosis process, which enables clinicians to initiate timely treatment strategies and tailor interventions based on individual patient profiles, ultimately optimizing therapeutic outcomes.

## Author contributions

**Conceptualization:** Azamossadat Hosseini.

**Data curation:** Hassan Emami, Mohammad Ali Nahayati.

**Formal analysis:** Hassan Emami.

**Funding acquisition:** Mohammad Ali Nahayati.

**Investigation:** Arash Roshanpoor.

**Methodology:** Arash Roshanpoor.

**Supervision:** Azamossadat Hosseini, Hassan Emami.

**Validation:** Arash Roshanpoor.

**Visualization:** Arash Roshanpoor.

**Writing – original draft:** Reza Darrudi, Azamossadat Hosseini, Hassan Emami, Mohammad Ali Nahayati.

**Writing – review & editing:** Reza Darrudi, Azamossadat Hosseini, Hassan Emami, Arash Roshanpoor, Mohammad Ali Nahayati.

## Supplementary Material


