# Supplementary material for: Artificial intelligence in the diagnosis of multiple sclerosis using brain imaging modalities: A systematic review and meta-analysis of algorithms
Source: Medicine (Baltimore). 2025 Sep 19;104(38):e44493. doi: 10.1097/MD.0000000000044493 (PMC12459500; doi:10.1097/MD.0000000000044493)
Supplement: Supplementary file 1 [file medi-104-e44493-s001.docx]

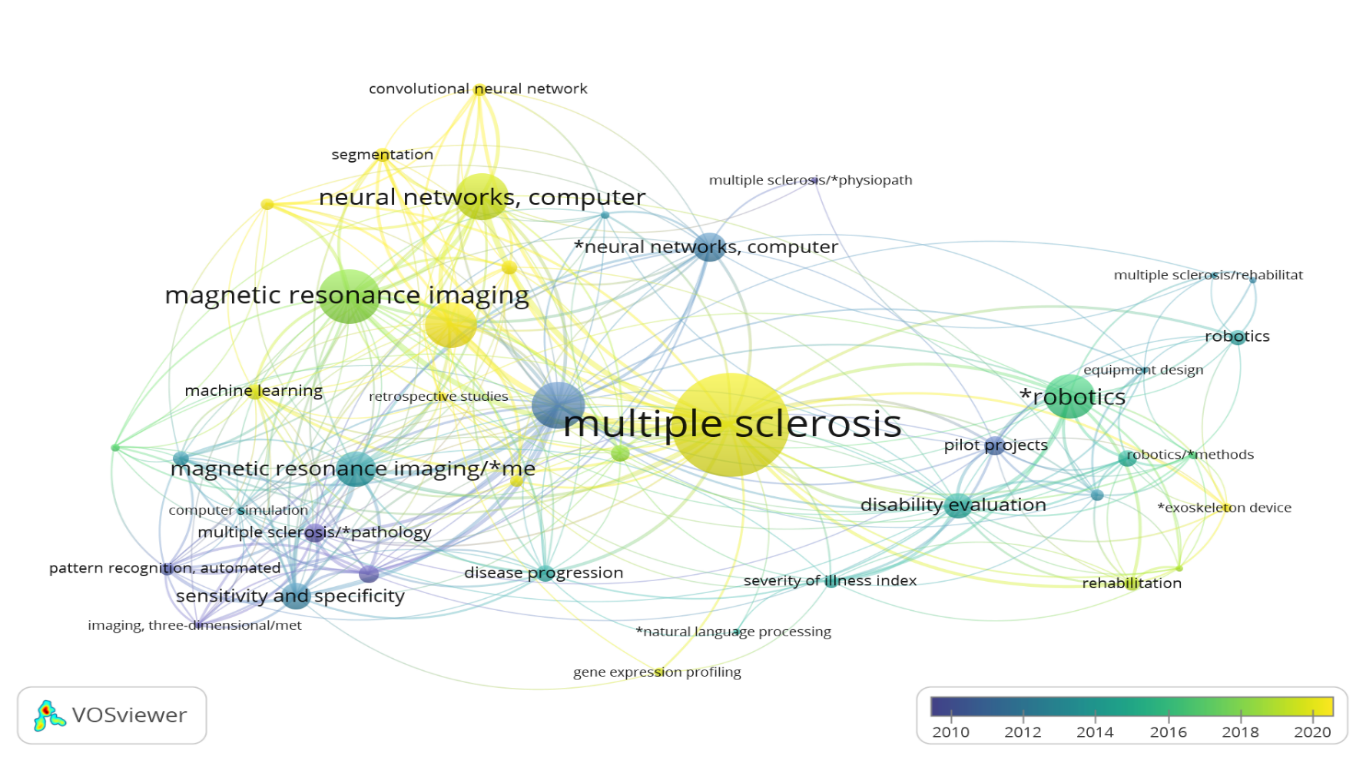


**Supplementary** **Figure 1.** The bibliometrics network map of artificial intelligence and MS

**Supplementary Figure 2.** Variety used AI methods in the included studies
